# Topical Nitroglycerin for Radial Access Optimization: Supporting Vascular Access in Patients at Risk for Acute Heart Failure

**DOI:** 10.3390/medicina61061016

**Published:** 2025-05-29

**Authors:** Adrian Sebastian Zus, Simina Crișan, Silvia Luca, Daniel Nișulescu, Mihaela Valcovici, Oana Pătru, Mihai-Andrei Lazăr, Cristina Văcărescu, Dan Gaiță, Constantin-Tudor Luca

**Affiliations:** 1 Cardiology Department, “Victor Babes” University of Medicine and Pharmacy, 300041 Timisoara, Romania; adrian.zus@umft.ro (A.S.Z.); silvia.luca0@student.umft.ro (S.L.); mihaela.valcovici@umft.ro (M.V.); oana.patru@umft.ro (O.P.); lazar.mihai@umft.ro (M.-A.L.); cristina.vacarescu@umft.ro (C.V.); dan.gaita@umft.ro (D.G.); constantin.luca@umft.ro (C.-T.L.); 2 Institute of Cardiovascular Diseases Timisoara, 300310 Timisoara, Romania; daniel.nisulescu@umft.ro; 3 Research Center of the Institute of Cardiovascular Diseases Timisoara, 300310 Timisoara, Romania; 4 Department of Histology, Faculty of Medicine, Vasile Goldis Western University of Arad, 310025 Arad, Romania

**Keywords:** topical nitroglycerine, radial artery spasm, transradial access, coronary angiography, heart failure, nitric oxide, radial artery puncture

## Abstract

*Background and Objectives*: Radial artery spasm (RAS) is a frequent complication during invasive angiography using the transradial approach, leading to patient discomfort and procedural challenges. While intra-arterial nitroglycerine (NTG) effectively reduces RAS after sheath insertion, preprocedural prevention strategies are limited. This study evaluates the efficacy of topical NTG in improving radial artery puncture success and reducing RAS incidence. *Materials and Methods:* In a randomized, double-blind single-center study 100 patients undergoing angiography were pretreated with either topical NTG or placebo. Outcomes assessed included RAS incidence, radial artery puncture success, number of attempts, procedural duration, patient discomfort, and complications. RAS was evaluated angiographically and clinically, with additional subgroup analyses for diabetic and smoking patients. *Results*: Topical NTG significantly reduced RAS incidence (53.2% vs. 73.6%; *p* = 0.0349) and increased radial puncture success on the first attempt (89.4% vs. 77.4%; *p* = 0.0488). Diabetic patients particularly benefited from NTG application, with lower RAS rates (36.4% vs. 76.2%; *p* = 0.0296). No significant differences were observed in procedural duration, patient discomfort, or complication rates between groups. The placebo group demonstrated a higher incidence of diffuse RAS (*p* = 0.0109). *Conclusions:* Preprocedural topical NTG application is a safe, non-invasive intervention that improves radial artery access success and reduces RAS, especially in high-risk subgroups such as diabetics. These findings support its potential as a procedural optimization tool in cardiovascular interventions, particularly in patients with heart failure, who often require repeated and reliable vascular access.

## 1. Introduction

Heart failure remains a major global health burden, with growing prevalence and significant morbidity and mortality. It is estimated that 1–2% of the world population is affected by heart failure, the most common cause being ischemic heart disease [1]. Chronic coronary syndrome represents the baseline upon which acute coronary syndrome develops, the latter putting patients at high risk of developing acute heart failure [2]. Clinical outcomes in this scenario are improved by invasive management [3], which is also recommended by current guidelines in the management of chronic coronary syndrome in patients with high-risk characteristics (large ischemic area, high-risk anatomical distribution of coronary disease), or who are still symptomatic under medical therapy alone [4].

The transradial approach (TRA) for coronary angiography and percutaneous coronary intervention has become the preferred vascular access route due to its association with lower bleeding risk, faster recovery, and even mortality benefits, particularly in high-risk patients such as those with acute heart failure or hemodynamic instability [5,6].

However, the utility of TRA is frequently limited by radial artery spasm (RAS), a complication arising from the artery’s rich smooth muscle content and sympathetic innervation [7,8]. RAS can appear in up to a quarter of all patients [9], and may lead to significant patient discomfort, longer procedure times, difficult catheter manipulation, higher radiation exposure, and increased contrast use [10].

Currently, strategies to mitigate RAS include sheath downsizing, pharmacological vasodilation, and pre-procedural sedation [9]. Intra-arterial NTG, often administered after sheath insertion, is a well-established agent for spasm relief [11,12]. However, it cannot prevent spasm that occurs prior to sheath insertion—a critical phase where pain, anxiety, and environmental factors may trigger arterial constriction.

Building on the vasodilatory potential of topical agents, this study evaluates whether preprocedural topical application of NTG can improve first-attempt radial artery puncture success and reduce RAS incidence. The study also explores the potential benefits in high-risk subgroups, such as patients with diabetes, who frequently present with endothelial dysfunction and are over-represented among those with heart failure. Our findings may support a simple, non-invasive intervention to optimize radial access—a cornerstone of cardiovascular care in the heart failure population.

## 2. Materials and Methods

### 2.1. Study Design

During the period of April 2023–June 2023, 100 consecutive patients who underwent invasive angiography using the radial artery approach (deemed a suitable access site by operating physician) were enrolled and randomized to either pretreatment with a topical cream 0.4% NTG, or application of a gel that contained no active substances. Sample size calculation was performed using MedCalc Statistical Software (version 22; MedCalc Software Ltd., Ostend, Belgium). Assuming a type I error (α) of 0.05 and a type II error (β) of 0.20 (80% power), we estimated proportions of 40% in group 1 and 70% in group 2, with an expected group size ratio of 1:1. This calculation indicated that 84 participants (42 in group 1 and 42 in group 2) would be required to detect a statistically significant difference between groups using a Chi-squared test. Randomization was done using a computer program designed to randomly produce either the digit 1 or 0, with 1 meaning the patient was assigned to nitroglycerine cohort and 0 to placebo cohort. The assigned nurse that handled the randomization also administered the patient’s treatment. The NTG or placebo cream was applied 20–40 min before radial artery puncture by the assigned nurse. Both creams were similar in appearance. The study was designed as a randomized, double-blinded single-center study, with neither the patient nor the operator aware of the treatment allocation.

### 2.2. Enrollment, Procedure Aspects, and Data Collection

**Figure 1:**
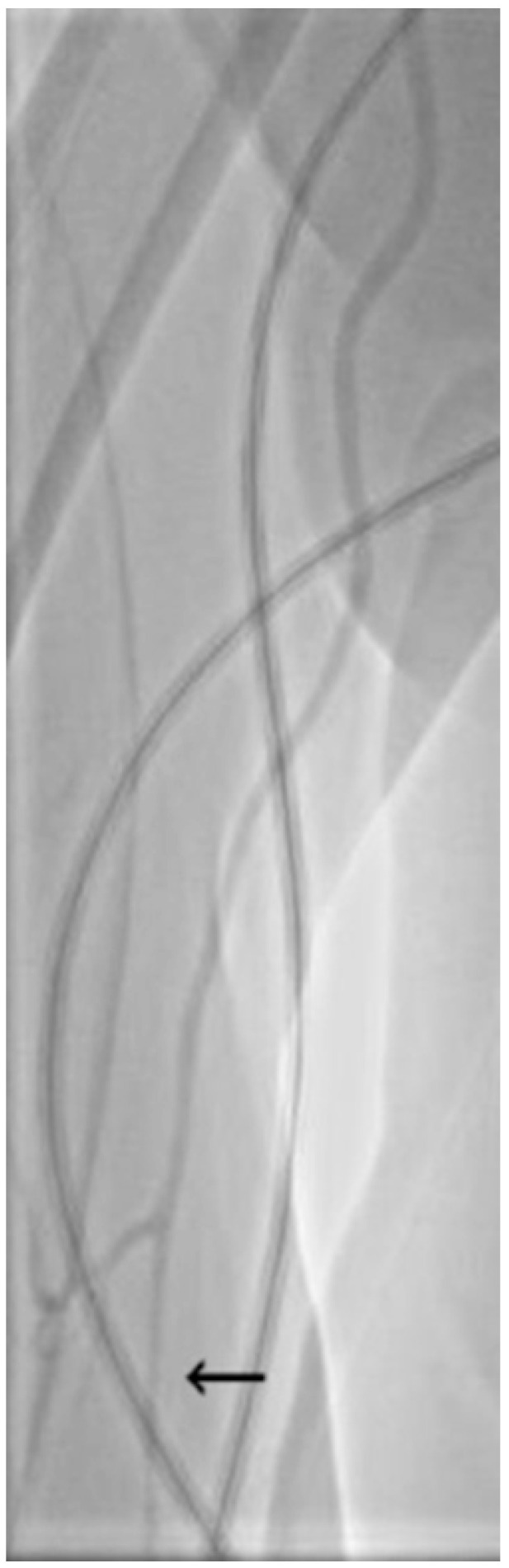
Angiogram of a radial artery that shows more than 50% diameter stenosis in the proximal part (highlighted by black arrow), consistent with RAS.

Patients had indication of either elective or emergency coronary artery or peripheral artery angiography and had no contraindication to radial artery approach. Both right and left radial arteries were used, depending on operator judgment, while the distal radial approach was not utilized in any case. The operator assessed the radial artery pulse quality by manual palpation before puncture. Standard local subcutaneous anesthesia with 5 mL of 2% lidocaine solution was performed. A visual analog scale was used to judge patient discomfort related to the puncture and puncture site at several times during the procedure, with the worst pain level being documented (scale of 0 to 10, where 0 is no pain, and 10 is worst possible pain). The key objective and primary endpoint were assessing spasm of the radial artery, which was judged both subjectively by the operator by judging the grade of resistance felt during manipulation of the catheters, and objectively by injection of contrast dye into the radial artery through the introducer sheath immediately after it had been placed inside the radial artery. Spasm was considered to be narrowing of the radial artery lumen by more than 50%. Diffuse spasm was considered to be spasm of a segment of radial artery greater than 2 cm, or more than two different sites of spasm. The diameter of the radial artery was also measured on the radial angiograms (Figure 1) and was indexed to the patient’s body surface area (BSA). After sheath insertion and radial artery measurement, the operator could administer intra-arterial NTG at his discretion throughout the procedure.

**Figure 2:**
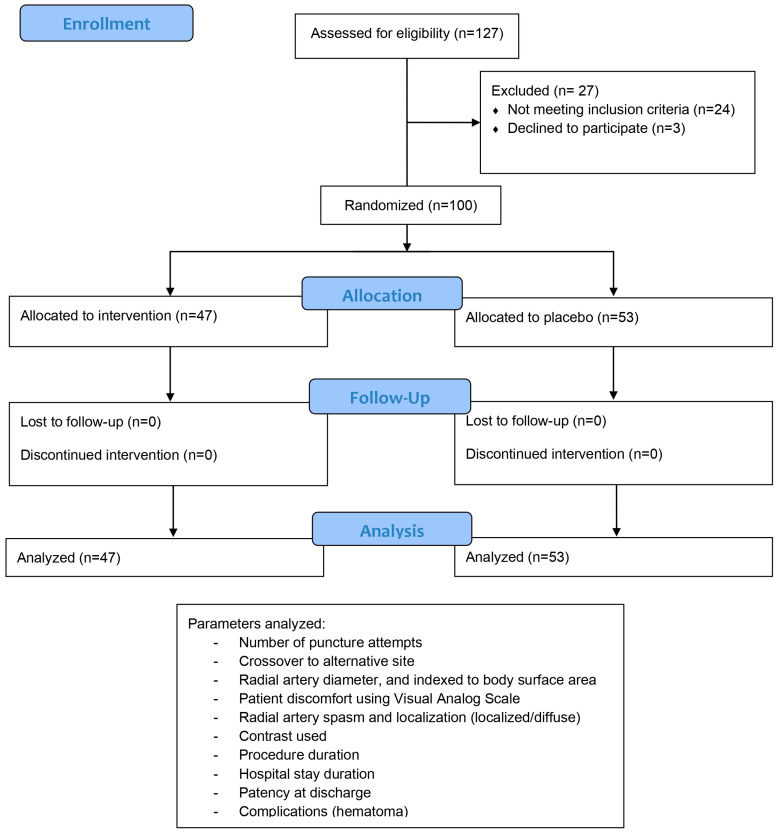
Study protocol pathway describing number of enrolled patients, number of patients in each randomized arm, and parameters that were pleasured for each patient.

Other data that were included were the number of attempts that led to successful puncture and sheath placement, procedure duration (including angioplasty if needed), contrast dye used, and hospital stay duration. Patients were screened for cardiovascular risk factors such as smoking, diabetes mellitus, dyslipidemia, hypertension, and obesity, and heart failure was assessed by measuring left ventricular (LV) ejection fraction by transthoracic echocardiography, and functional categorization (NYHA class). Procedural anticoagulation was performed using 5000 units of unfractioned heparin for diagnostic procedures and 70 units/kg for angioplasties. At the end of the procedure, after sheath extraction, hemostasis was achieved using a compressive dressing that was left in place for ~12 h. Bleeding complications at the site of puncture were noted (presence of local hematoma), and ultrasound screening at discharge was used to check postprocedural radial artery patency (Figure 2). Radial artery occlusion (RAO) was defined as absence of color and Doppler signal in the radial artery at the puncture site.

The radial sheaths used were 6 French, and the operator had the freedom to use any size of catheters he considered, most often choosing 6 French for both diagnostic angiography and percutaneous intervention. No preprocedural patient sedation was used, and the cath lab temperature was similar for all cases.

Statistical analysis was performed using IBM SPSS Statistics version 20.0 software for Windows with a significant *p* < 0.05. We used descriptive statistics, figures, and tables to summarize our findings. Results for targeted variables were presented using descriptive statistics (mean, standard deviation, range, median, and associated inter-quartile range) for continuous data, and counts with associated percentages for categorical data. Independent samples *t*-tests were used to analyze differences in means for continuous variables, while differences between categorical variables were examined by Chi-squared tests. Categorical data are presented as counts (percentages).

## 3. Results

Of the 100 patients who met the inclusion criteria (out of 127 total patients considered for inclusion), 47 were randomized to the nitroglycerine cohort and 53 to the placebo cohort. Collected patients’ data are summarized and then compared by study arm in Table 1, Table 2, Table 3 and Table 4, with subgroup analysis for diabetics and smokers presented in Table 5. Overall, there were more than twice as many males as females enrolled, with about a third of all patients smokers, one-third diabetics, and almost all dyslipidemic. Approximately half of patients were chronic and half were acute cases. Baseline characteristics, including age, body mass index, heart failure class, ejection fraction, radial pulse quality, and blood pressure were similar between the groups. Two-thirds of all enrolled patients had radial artery spasm confirmed angiographically. Radial artery diameter after sheath placement was not different in the two subgroups, and pain reported was also of similar intensity (on average around 4 on the Visual Analog Scale). Crossover from the radial to the femoral or brachial artery occurred due to radial artery spasm in 4/53 (7.5%) cases from the placebo cohort, with a *p* value of borderline statistical significance (0.0546). No harm or unintended results were reported.

**Figure 3:**
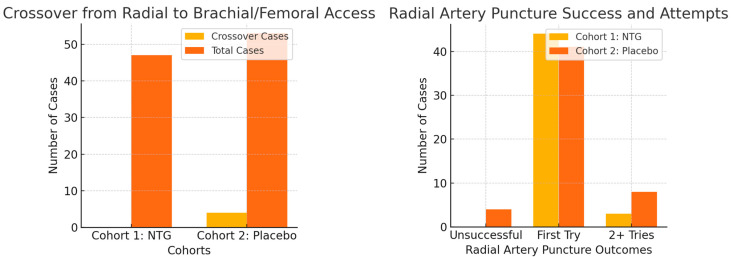
Radial artery puncture success was increased by topical NTG (*p* = 0.04), and crossover rates were also lower in this group (*p* = 0.05).

**Figure 4:**
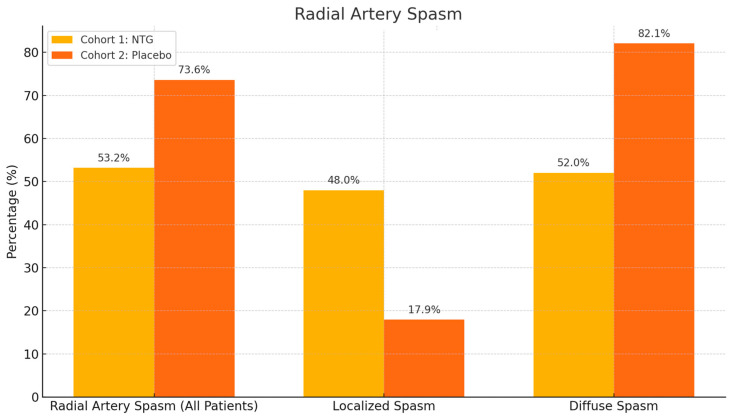
RAS incidence was lower in NTG patients than in the placebo group (*p* = 0.02 for spasm occurrence), and when spasm did occur, it was more frequently localized and not diffuse (*p* = 0.01 for difference in localized/diffuse spasm).

Radial artery puncture was more successful in the NTG group, requiring fewer attempts (Figure 3). First-attempt puncture was successful in 44 out of 47 patents in the NTG group, with no failures, while only 41 out of 53 first-try punctures were successful in the placebo group, with four failures leading to crossover to alternative puncture sites, with a statistically significant *p* value of 0.0488. The overall incidence of spasm was significantly lower in the NTG group—25/47 (53.2%) compared to the placebo group—39/53 (73.6%), with a *p* value of 0.02 (Figure 4). Additionally, the incidence of diffuse spasm was significantly higher in the placebo group (32/39—82.1%) compared to the NTG group (13/25—52.0%), with a *p* of 0.0109. Other outcomes, such as complications, procedure length, contrast used, and hospital stay, did not show any difference between the two cohorts. We did, however, note a trend towards less postprocedural radial artery occlusion (90.6% compared to 97.9% patency, but with *p* above significance level).

**Figure 5:**
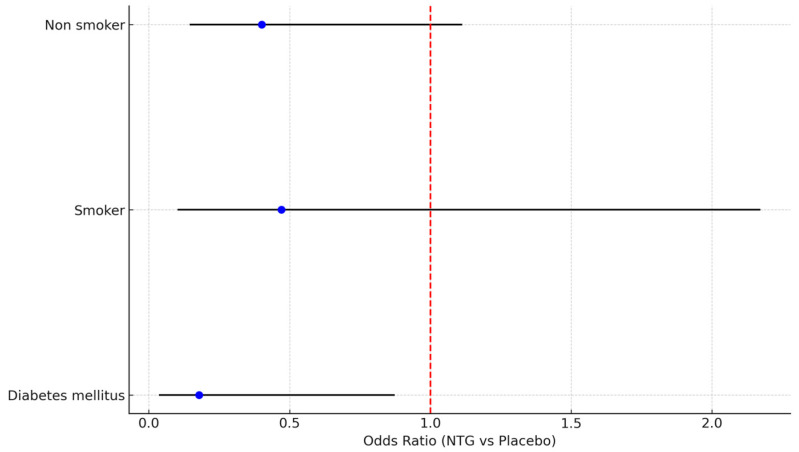
Forest plot describing effect of NTG on RAS in subgroups of diabetics, smokers, and non-smokers.

Among diabetic patients, spasm incidence was notably reduced with NTG (4/11—36.4% vs. 16/21—76.2% in the placebo group, *p* = 0.02). While no difference in RAS was observed in smokers, a trend towards less RAS was evident in non-smokers (Figure 5).

## 4. Discussion

Our findings demonstrate that topical NTG significantly increases radial artery puncture success while reducing the incidence of RAS, especially in diabetic patients. This is likely due to decreased nitric oxide bioavailability, which is often compromised in patients with diabetes and cardiovascular comorbidities [13]. As diabetic patients fail to synthetize enough nitric oxide, an exogenic source, such as the one we administered, has the potential to compensate for this and induce vasodilation of the radial artery. Results underscore the potential importance of NTG in managing procedure-related vasospasm, confirming the results of a previous study that similarly showed reduction in radial artery spasm following preprocedural application of topical NTG, but with the addition of topical lidocaine to both the NTG and the control group [14].

Interestingly, the limited benefit observed in smokers may be attributed to their baseline endothelial dysfunction and increased oxidative stress, which can impair vasodilatory responses to nitric oxide [15]. In contrast, non-smokers—potentially with preserved endothelial function—may exhibit more pronounced responses to topical NTG, further supporting its utility in selected populations.

Although the reduction in RAS did not translate into shorter procedural durations or reduced patient discomfort, this may reflect the routine intra-arterial NTG administration used as rescue therapy in both groups. Nonetheless, preventing early RAS through preprocedural topical vasodilation remains valuable, particularly in high-risk groups where even minor procedural complications can impact outcomes—heart failure patients often are at high bleeding risk due to antithrombotic therapy for associated pathologies (coronary artery disease, atrial fibrillation), and femoral access, especially in the acute setting, increases risk of adverse cardiovascular events [16].

Importantly, we observed a trend toward improved radial artery patency at discharge in the NTG group. While not statistically significant, this observation merits further study, as RAO—though often asymptomatic—can preclude future access. In patients with heart failure, who frequently require repeated vascular access for coronary evaluation, electrophysiology procedures, or hemodynamic monitoring, preserving the radial artery is especially relevant [17].

Our study builds upon prior work demonstrating that NTG and lidocaine can increase radial artery diameter [18,19], and we extend this by showing a clinically meaningful reduction in RAS with topical NTG alone. Notably, our angiographic measurements did not reveal significant changes in radial diameter, suggesting that NTG’s anti-spastic effects may be functional rather than structural in some cases.

Although a general reduction in spasm was clearly observed, this did not lead to shorter procedure times or less patient discomfort, but this could be explained by the fact that the operator had the possibility of further intra-arterial NTG administration at any point during the procedure, which would have been performed in those patients who had documented RAS on angiogram. While out study did not prove a benefit in reducing pain experienced by patients, evidence exists that topical medication could be effective in this regard [20].

Existing data suggest that intra-arterial NTG does not reduce the rate of postprocedural radial artery occlusion [21], but, considering the trend towards better patency observed in our study, larger patient enrollment in future studies might help shed light on a potential supplemental benefit of topical NTG, as RAO occurs in up to 10% of cases [16], and while it is often clinically insignificant, it does take away a potential access site for future procedures. Larger studies could potentially demonstrate additional benefits of topical NTG administration prior to radial artery invasive procedures, as definite conclusions are hard to draw due to the limited number of studies and small number of patients enrolled [22].

From a heart failure perspective, optimizing vascular access—especially in fragile or comorbid patients—is crucial. TRA is the preferred route in these patients due to its safety profile, but complications, like RAS, remain a barrier. The application of topical NTG, which is inexpensive and easy to apply, represents a simple, cost-effective intervention that could streamline diagnostic and interventional procedures in this population.

No adverse events were noted, but targeted studies might provide insight into more vulnerable patients (such as those with severe aortic stenosis), which might more easily develop side-effects such as hypotension, headache, or worsening heart failure. Although intraprocedural patients’ discomfort was not improved, patient satisfaction queries could be used to assess feedback and overall experience during angiography.

Limitations to our study include single-center and single-operator design, and lack of long-term follow-up, the latter important in determining potential effects of repeated NTG cream application. Moreover, investigating alternative vasodilators—such as calcium channel blockers or phosphodiesterase inhibitors—or different doses of NTG-based topical agents, and potentially combination creams containing several drugs, may provide additional tools to enhance procedural success and safety in the expanding field of heart failure intervention.

## 5. Conclusions

Topical nitroglycerine significantly improves radial artery puncture success and reduces the incidence of radial artery spasm, particularly in high-risk subgroups such as diabetic patients. As TRA becomes increasingly preferred in the management of cardiovascular diseases, including in patients with heart failure, minimizing access-related complications is critical. This study highlights a simple non-invasive strategy that may enhance procedural safety, preserve vascular integrity, and improve patient experience during coronary angiography. Further large-scale studies are warranted to confirm these findings and explore the potential of topical vasodilators as adjuncts in the interventional management of heart failure and related conditions.

## Figures and Tables

**Table 1:** Data presentation of the two cohorts. It was guided by the D’Agostino–Pearson omnibus test for normality. Variables with a normal distribution are reported as mean ± SD; non-normal variables are reported as median [inter-quartile range]. Minimum and maximum values are provided for all continuous variables.

All Patients *N* = 100	Median	Median CI	Median SD	Minimum	Maximum
Height (centimeters)	168.27	166.37 to 170.16	9.57	150	190
Procedure duration (minutes)	26.43	22.57 to 30.30	19.27	3	133
Contrast used during procedure (milliliters)	147.90	126.26 to 169.55	107.96	50	650
Pain score (based on Visual Analog Scale)	3	3.0 to 4.0	2.27	0	9
Hospital stay duration (days)	3.38	2.90 to 3.85	2.39	1	19
**All patients** ***N* = 100**	**Mean**	**Mean CI**	**Mean SD**	**Minimum**	**Maximum**
Age (years)	62	60 to 67	11.23	32.00	88.00
Body mass index (kilograms/meters squared)	28.07	27.56 to 29.30	4.77	16.89	40.62
Height (centimeters)	170	166 to 170	9.57	150	190
Weight (kg) LV Ejection fraction (%) NYHA class	80 45 2	77.44 to 83.27 40 to 50 2.0 to 2.0	14.94 11.15 0.67	46 15 1	118 65 4
Mean blood pressure (mmHg)	115	110 to 120	17.75	65	165
Radial artery diameter indexed to body surface area (millimeters/meters squared)	1.25	1.17 to 1.28	0.27	0.75	2.30

**Table 2:** Categorical values and their percentages.

All Patients *N* = 100	*N* = 100 (%)
Sex	male = 71/100 (71%)
Presentation	Acute = 44/100 (44%)
Diabetes mellitus	32/100 (32%)
Dyslipidemia	95/100 (95%)
Smoking	36/100 (36%)
Hypertension	80/100 (80%)
Previous radial intervention	17/100 (17%)
Radial artery pulse grade	1/100—absent (1%) 34/100—weak (34%) 48/100—good (48%) 17/100—very good (17%)
Radial artery puncture success and tries	4/100—unsuccessful (4%) 83/100—first try (83%) 13/100—2 or more tries (13%)
Crossover from radial to brachial/femoral access due to failed puncture	4/100 (4%)
Radial artery spasm	63/100 (63%)
Localized or diffuse spasm	Localized = 20/63 (31.7%) Diffuse = 43/63 (69.3%)
Radial artery patency at discharge	95/100 (95%)
Hematoma at puncture site	19/100 (19%)

**Table 3:** Comparison of numeric values for the two cohorts. Variables that met normality assumptions are shown as mean ± SD and were compared between cohorts with an unpaired, two-tailed Student *t*-test. Variables that were non-normal are presented as median [IQR] and were compared with the two-tailed Mann–Whitney U test. A two-sided α = 0.05 was considered statistically significant; the 95% confidence interval (CI) for the between-group difference is provided for each comparison.

All Patients *N* = 100	Cohort 1: NTG *N* = 47 Median	Cohort 1 Median CI	Cohort 1 IQR	Cohort 2: Placebo *N* = 53 Median	Cohort 2 Median CI	Cohort 2 IQR	*p*	CI
Height (centimeters)	170	166.63 to 173.68	165 to 175	168	163.00 to 170.00	160.75 to 175.00	*p* = 0.1168	−6.0000 to 0.0000
Procedure duration (minutes)	29	15.00 to 32.00	11.00 to 40.00	20	15.00 to 26.96	11.00 to 30.75	*p* = 0.3276	−10.0000 to 2.0000
Contrast used during procedure (milliliters)	150	50 to 200	50 to 200	100	71 to 150	50 to 200	*p* = 0.8069	−50.0000 to 20.0000
Pain score (based on Visual Analog Scale)	4	3 to 5	2 to 6	3	3 to 4	2 to 5.5	*p* = 0.6489	−1.0000 to 1.0000
Hospital stay duration (days)	3	2.31 to 3.68	2 to 5	3	2.89 to 3.00	2 to 4	*p* = 0.6136	−1.0000 to 0.0000
**All patients** ***N* = 100**	**Cohort 1: NTG** ***N* = 47** **Mean**	**Cohort 1 Mean CI**	**Cohort 1 Mean SD**	**Cohort 2: Placebo *N* = 53 Mean**	**Cohort 2 Mean CI**	**Cohort 2 Mean SD**	***p***	**CI**
Age (years)	61.08	57.60 to 64.56	11.86	64.52	61.63 to 67.42	10.49	*p* = 0.1267	−1.02 to 7.91
Body mass index (kilograms/meters squared)	28.56	27.33 to 29.80	4.20	28.94	27.48 to 30.39	5.2709	*p* = 0.6992	−1.5099 to 2.2552
Weight (kg)	83.04	78.879 to 87.20	14.17	80.90	76.587 to 85.22	15.66	*p* = 0.4757	−8.0596 to 3.7859
LV Ejection fraction (%)	45.38	42.41 to 48.35	10.11	43.53	40.22 to 46.84	12.02	*p* = 0.409	−2.54 to 6.25
NYHA class	1.94	1.77 to 2.10	0.57	2.06	1.85 to 2.26	0.74	*p* = 0.551	−2.0 to 1.0
Mean blood pressure (mmHg)	114.04	109.298 to 118.78	16.15	114.52	109.23 to 119.82	19.20	*p* = 0.8922	−6.6082 to 7.5797
Radial artery diameter (millimeters)	2.44	2.30 to 2.57	0.44	2.46	2.31 to 2.62	0.53	*p* = 0.7818	−0.1718 to 0.2276
Radial artery diameter indexed to body surface area (millimeters/meters squared)	1.24	1.17 to 1.31	0.23	1.30	1.21 to 1.39	0.31	*p* = 0.352	–0.85 to 0.68

**Table 4:** Categorical values for the two cohorts. Bold: *p* values that are significant (<0.05).

All Patients *N* = 100	Cohort 1: NTG *N* = 47	Cohort 2: Placebo *N* = 53	*p*
Sex	11/47 (23.4%) female 36/47 (76.6%) male	18/53 (34%) female 35/53 (66.0%) male	*p* = 0.2479
Acute/chronic presentation	23/47 (48.9%)	21/53 (60.4%)	*p* = 0.3515
Diabetes mellitus	11 out of 47 (25.5%)	21 out of 53 (39.6%)	*p* = 0.0842
Dyslipidemia	45/47 (95.7%)	50/53 (94.3%)	*p* = 0.7489
Smoking	32/47 no (68.1%) 15/47 yes (31.9%)	32/53 no (60.4%) 21/53 yes (39.6%)	*p* = 0.4229
Hypertension	35/47 (74.5%)	44/53 (83%)	*p* = 0.2972
Previous radial intervention	11/47 (23.4%)	6/53 (11.3%)	*p* = 0.1102
Radial artery pulse grade	0 absent 12 weak (25.5%) 27 good (57.4%) 8 very good (17%)	1 absent (1.9%) 22 weak (41.5%) 21 good (39.6%) 9 very good (17%)	*p* = 0.2208
Radial artery puncture success and tries	0/47 unsuccessful 44/47 (89.4%) first try 3/47 (10.6%) 2 or more tries	4/53 (7.5%) unsuccessful 41/53 (77.4%) first try 8/53 (15.1%) 2 or more tries	***p* = 0.0488**
Crossover from radial to brachial/femoral access	0/47 (0%)	4/53 (7.5%)	***p* = 0.0546**
Radial artery spasm	25/47 (53.2%)	39/53 (73.6%)	***p* = 0.0349**
Localized or diffuse spasm	12/25 localized (48.0%) 13/25 diffuse (52.0%)	7/39 (17.9%) localized 32/39 diffuse (82.1%)	***p* = 0.0109**
Radial artery patency at discharge	46/47 (97.9%)	48/53 (90.6%)	*p* = 0.1266
Hematoma at puncture site	9/47 (19.1%)	10/53 (18.9%)	*p* = 0.9716

**Table 5:** Sub analysis of cohorts (diabetics, smokers, and non-smokers). Bold: *p* values that are significant (<0.05).

	Cohort 1 NTG with Diabetes Mellitus *N* = 11	Cohort 2 Placebo with Diabetes Mellitus *N* = 21	*p*
Radial artery spasm	4/11 (36.4%)	16/21 (76.2%)	***p* = 0.0296**
	**Cohort 1 NTG smoker** ***N* = 15**	**Cohort 2** **Placebo** **smoker** ***N* = 21**	
Radial artery spasm	10/15 (66.6%)	17/21 (80.9%)	*p* = 0.5580
	**Cohort 1 NTG Non smoker** ***N* = 32**	**Cohort 2** **Placebo** **Non smoker** ***N* = 32**	
Radial artery spasm	15/32 (46.8%)	22/32 (68.7%)	*p* = 0.1290

## Data Availability

The original contributions presented in this study are included in the article. Further inquiries can be directed to the corresponding author.

## Abbreviations

The following abbreviations are used in this manuscript:

TRA	Transradial access
RAS	Radial artery spasm
NTG	Nitroglycerine
BSA	Body surface area
LV	Left Ventricular
NYHA	New York Heart Association
CI	Confidence interval
SD	Standard deviation
IQR	Interquartile range
RAO	Radial artery occlusion
